# Downregulation of Sp1 by Minnelide leads to decrease in HSP70 and decrease in tumor burden of gastric cancer

**DOI:** 10.1371/journal.pone.0171827

**Published:** 2017-02-13

**Authors:** Nivedita Arora, Osama Alsaied, Patricia Dauer, Kaustav Majumder, Shrey Modi, Bhuwan Giri, Vikas Dudeja, Sulagna Banerjee, Daniel Von Hoff, Ashok Saluja

**Affiliations:** 1 Div. of Basic and Translational Research Dept. of Surgery University of Minnesota, Minneapolis MN, United States of America; 2 Div. of Surgical Oncology Dept. of Surgery University of Miami, Miami, FL, United States of America; 3 TGen/Virginia G. Piper Cancer Ctr, Suite 600, Phoenix, AZ United States of America; University of Navarra, SPAIN

## Abstract

**Background:**

Gastric cancer is the third leading cause of cancer related mortality worldwide with poor survival rates. Even though a number of chemotherapeutic compounds have been used against this disease, stomach cancer has not been particularly sensitive to these drugs.

In this study we have evaluated the effect of triptolide, a naturally derived diterpene triepoxide and its water soluble pro-drug Minnelide on several gastric adenocarcinoma cell lines both as monotherapy and in combination with CPT-11.

**Methods:**

Gastric cancer cell lines MKN28 and MKN45 were treated with varying doses of triptolide in vitro. Cell viability was measured using MTT based assay kit. Apoptotic cell death was assayed by measuring caspase activity. Effect of the triptolide pro-drug, Minnelide, was evaluated by implanting the gastric cancer cells subcutaneously in athymic nude mice.

**Results:**

Gastric cancer cell lines MKN28 and MKN45 cells exhibited decreased cell viability and increased apoptosis when treated with varying doses of triptolide in vitro. When implanted in athymic nude mice, treatment with Minnelide reduced tumor burden in both MKN28 derived tumors as well as MKN45 derived tumors. Additionally, we also evaluated Minnelide as a single agent and in combination with CPT-11 in the NCI-N87 human gastric tumor xenograft model.

**Conclusion:**

Our results indicated that the combination of Minnelide with CPT-11 resulted in significantly smaller tumors compared to control. These studies are extremely encouraging as Minnelide is currently undergoing phase 1 clinical trials for gastrointestinal cancers.

## 1.Background

Gastric cancer is the fifth most common cancer type worldwide with 951000 new cases diagnosed in 2012 [1]. Asian countries bear the brunt of the disease with the rate of new cases being 4 times higher than in Africa [1]. The five-year survival for stage 1A gastric cancer is about 71% whereas that for advanced stages (stage III and stage IV), it is less than 20% (www.cancer.org). In the US alone, it is estimated that about 24590 cases of gastric cancer will be diagnosed in 2015 and about 10720 people will succumb to this disease [2]. Most cases are diagnosed at advanced stages of the disease in this country resulting in an average 5 year survival of about 29% [2].

Triptolide, a diterpene triepoxide derived from a Chinese herb, *Tripterygium Wilfordii* has been studied as an anti-leukemic agent, and a therapy for rheumatoid arthritis before its efficacy against solid tumors was explored. It has been shown to exhibit antitumor efficacy against pancreatic adenocarcinoma [3], breast cancer, neuroblastoma [4], colon cancer [5] and osteosarcoma [6]. Since the clinical utility of triptolide was restricted owing to its limited solubility, a water-soluble pro-drug was developed at the University of Minnesota [7]. Minnelide is currently in Phase 1 clinical trial against advanced GI malignancies.

Aberrant activation of oncogenes and tumor suppressor genes often contribute to the aggressiveness of gastric cancer. In addition, multiple growth factors and their receptors play an active role in gastric cancer growth progression. These molecular changes are often regulated by re-wired signaling pathways that are influenced by multiple internal and external factors [8, 9]. Transcription factors play an integral role in this process. Sp1 is a well-characterized, sequence-specific, DNA-binding protein that is important in the transcription of many cellular and viral genes containing GC boxes in their promoters [10]. Previous studies have shown that abnormal Sp1 activation might augment the growth and metastatic potential of tumor cells through over-expression of many Sp1 downstream genes. The role of Sp1 as an essential transcription factor for many genes regulating cell growth, angiogenesis and survival has been proved in pancreatic cancer [11,12].

The mechanism of action of triptolide/Minnelide remains elusive. In pancreatic cancer, triptolide decreases the expression and activity of O-GlcNAc transferase (OGT), which leads to inhibition of nuclear translocation of Sp1 (Specificity Protein 1) transcription factor. Sp1 is responsible for the transcription of many pro-survival genes including heat shock protein 70 (HSP70) and Heat shock factor 1 [13,14]. In cancer cells, it has been shown that overexpression of HSP70 is important for cell growth and survival as well as conferring resistance to apoptosis [15]. High levels of Sp1 and HSP70 in resected specimens of gastric adenocarcinoma have been shown to be associated with a poor prognosis [16, 17].

In the current study we have evaluated the efficacy of Minnelide on gastric cancer cell lines MKN45 and MKN28 both in vivo and in vitro. Additionally, we have also evaluated the effect of combining low doses of Minnelide with CPT-11, a chemotherapeutic agent approved for use in gastric adenocarcinoma. Our study demonstrates that triptolide is an efficacious agent for treatment of gastric cancer in vitro and, in the form of Minnelide, in vivo, both as a single agent as well as in combination. Our study further demonstrates that triptolide decreases the expression of Sp1 and as a result, HSP70 in two gastric adenocarcinoma cell lines leading to apoptosis.

## 2. Methods

### 2.1 Cell lines and reagents

We used human gastric cancer cell lines MKN45, MKN28 and NCI-N87. Cells were cultured in RPMI1640 media (Hyclone) supplemented with 10% fetal bovine serum (Hyclone) and 1% penicillin streptomycin antibiotic (Hyclone) solution. Cells were cultured in a humidified atmosphere with 5% CO_2_ and at 37 degrees Celsius and were passaged every 48 hours.

### 2.2 Triptolide treatment

Triptolide was dissolved in dimethyl sulfoxide to make a stock concentration of 1mg/dl and aliquots were stored at -20 degrees Celsius. For cell viabilities, caspase assays, RNA and protein extraction, cells were seeded in media supplemented with 10% serum. After 24 hours incubation, cells were treated with varying concentrations of triptolide in media supplemented with 10% FBS for defined times at 37 degrees. Untreated cells in serum containing media served as control.

### 2.3 Quantitative real time PCR

Quantitative realtime PCR for Sp1 and HSF1 was carried out using primers obtained from Qiagen (QuantiTect primer assay). Primers for HSP70 (forward Primer, ACCAAGCAGACGCAGATC; and reverse primer, CGCCCTCGTACACCTGGA) were synthesized by Life Technologies. RNA was isolated from the different cell lines according to the manufacturer's instructions using TRIzol (Invitrogen). One μg of total RNA was reverse transcribed using High Capacity cDNA Reverse Transcription Kit (Life Technologies) on the Peltier Thermal Cycler 200 (MJ Research). Realtime PCR was performed on a LightCycler II 480 (Roche) using Lightcycler 480 SYBR Green I (Roche). All data were normalized to the housekeeping 18S gene (18S QuantiTect primer assay, Qiagen).

### 2.4 Western blotting

Protein from treated and untreated cell lysates was estimated using the BCA protein estimation assay (Thermo Scientific). For Western blotting, anti-HSP70 antibody (Enzo life sciences), and anti-Sp1 antibodies (Cell Signaling) were used to check for levels of different proteins in the lysates. Actin was used as loading control. Actin antibody was obtained from Cell Signaling Technology.

### 2.5 Cell viability

Cell viability was determined using Dojindo Cell Counting Kit– 8 (Dojindo Molecular technologies) following the manufacturer’s instructions. 3000 cells were cultured in 100ul of medium in each well of a 96well plate and were allowed to grow for 24 hours before they were treated with triptolide. After treatment with triptolide for specified durations and concentrations, cell viability was assessed by incubation with 10ul of tetrazolium substrate for 1 hour at 37 degrees, followed by measurement of absorbance at 450nm.

### 2.6 Caspase 3/7 activity

Caspase-3/7 activity was analyzed by the Caspase-Glo luminescence based assays (Promega) according to the manufacturer’s protocol. Cells were seeded and treated with triptolide as described above. Then 100ul of appropriate Caspase-Glo reagents were added to each well. Caspase activity was normalized to the corresponding cell viability measurements.

### 2.7 Subcutaneous xenograft mouse model

All animal studies were approved by University of Minnesota and performed according to the institutional guidelines of the IACUC at the University of Minnesota.

Four to six week old female athymic nude mice (The Jackson laboratory) were injected subcutaneously with MKN28 or MKN45 cells in the right flank. Animals were housed in a specific pathogen free (SPF) facility at University of Minnesota. All animals were fed a standard normal diet (PMI Lab Diet, St. Louis, MO) *ad libitum* with free access to water.

For MKN28 cells, 2.5 million cells were injected per mouse whereas for MKN45 cells, 1 million cells were injected per mouse. Cells were suspended in Matrigel (BD Biosciences). The tumors were allowed to grow for two weeks after which mice were randomized to receive saline (control), 0.21 mg/kg Minnelide or 0.42 mg/kg Minnelide (n = 10 mice per group). Tumor volume was assessed twice weekly. Animals were monitored weekly for apparent signs of pain and discomfort. Tumor volume was calculated using the formula (long diameter * short diameter^2^ /2). Permissible tumor volume was 2cm^3^ according to the approved protocol. Mice were weighed weekly to assess for weight loss as a surrogate marker of drug toxicity and animal health. The experiment was terminated at 42 days for MKN28 cell line mice and at 21 days for MKN45 cell line mice. Experiments were performed and animals were sacrificed using CO_2_ chamber in accordance with the regulations of the Institutional Animal Care and Use Committee (IUCAC) of the University of Minnesota. Close attention was paid to humane endpoints during the course of all animal experiments.

For combination study, athymic nude mice were inoculated in the subcutaneous right flank with 0.1ml of a 1:1 Matrigel™: Media (BD Biosciences) mixture containing a suspension of NCI-N87 tumor cells (approximately 1 x 10^7^ cells/mouse). Ten days following inoculation, sixty mice with tumor volumes of 99–190 mm^3^ were randomized into six groups of ten mice each by random equilibration, each group with a mean tumor volume of approximately 150–151 mm^3^. Tumor volumes and body weights were recorded when the mice were randomized and were measured two times weekly thereafter. Gross observations were recorded daily. The study was ended on Day 40.

### 2.8 Terminal deoxynucleotidyl transferase–mediated dUTP nick end labeling (TUNEL) assay for in situ apoptosis

Paraffin-embedded xenograft tissue sections from treated mice were processed for terminal deoxynucleotidyl transferase–mediated dUTP nick end labeling (TUNEL) assay using In Situ Cell Death Detection Kit, Fluorescein (Roche) according to the manufacturer's instructions. Counterstaining for total cells was done with DAPI. Coverslips were applied and fixed with permount. Images were taken on a confocal microscope (Nikon) using a magnification of 20X for overview and for quantifying the images. 10 random field images were taken per tumor section slide. The images were quantified using ImageJ software (NIH) and the mean was calculated for the ratio of total TUNEL positive cells to total DAPI positive cells. TUNEL is a method of detecting DNA fragmentation resulting from apoptotic signaling cascades.

### 2.9 Overexpression of Sp1

Sp1 was overexpressd in MKN28 and MKN45 cell lines pCMV-Sp1 (Origene). Sp1 overexpressing cells were plated and treated with 100nM triptolide to assess viability as described above. Non-overexpressing cells served as control.

### 2.10 Statistical analysis

Data are presented as mean ± SEM of three separate experiments. The significance of the difference between the control and each individual experimental condition was analyzed by unpaired Student’s *t* test. A *P* < 0.05 was considered as a statistically significant difference.

## 3.Results

### 3.1 Triptolide induces apoptotic cell death in gastric cancer cells *in vitro*

**Fig 1:**
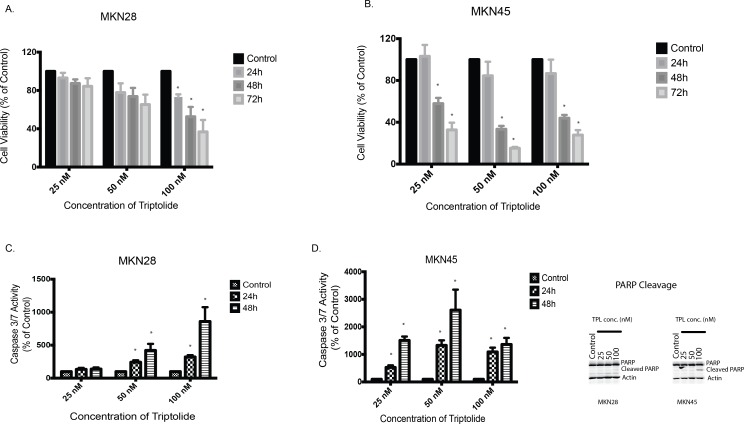
**A. Triptolide induces apoptotic cell death in gastric cancer cell line.** Treatment of moderately differentiated gastric adenocarcinoma cell line, MKN28 with triptolide decreases viability in a dose (25–100nM) as well as time (24–72 hours) dependent manner. Untreated cells served as control. Viability was assessed using CCK-8. **B.** Treatment of poorly differentiated gastric adenocarcinoma cell line, MKN45 with triptolide decreases viability in a dose (25–100nM) as well as time (24–72 hours) dependent manner. Untreated cells served as control **C.** Triptolide causes cell death in MKN28 cells via induction of apoptosis, as evidenced by a dose (25–100nM) as well as time (24–48 hours) dependent increase in caspase 3/7 activation. Untreated cells served as control. **D.** Triptolide causes cell death in MKN45 cells via induction of apoptosis, as evidenced by a dose (25–100nM) as well as time (24–48 hours) dependent increase in caspase 3/7 activation. Untreated cells served as control. E. Western blot showing cleaved PARP as a measure of apoptosis in MKN28 and MKN45 cells after treatment with indicated doses of triptolide. * indicates p value < 0.05 when compared to untreated.

Moderately differentiated cell line MKN28 and poorly differentiated cell line MKN45 isolated from the lymph node and liver metastases, respectively of gastric adenocarcinoma were used for our studies. To study the effect of triptolide on cell viability, MKN28 and MKN45 cells were treated with indicated doses of triptolide for 24, 48 or 72 hours *in vitro*. Both cell lines showed a time as well as dose dependent decrease in viability following treatment. 100nM of triptolide was able to decrease the viability of MKN28 cells to 73%, 52% and 37% of control at 24, 48 and 72 hours respectively (Fig 1A). For MKN45 cells, 50nM of triptolide was able to decrease the viability to 84%, 33% and 15% of control at 24, 48 and 72 hours respectively. (Fig 1B). To study if cell death was apoptotic, we assayed for cleaved caspase activity in gastric cancer cell lines treated with triptolide. Cleaved Caspase 3 is a measure of apoptotic cell death. Upon treatment with indicated doses of triptolide for 24 and 48 hours, Caspase 3/7 levels were estimated using untreated cells as control. For MKN28 cells, with 100nM of triptolide, Caspase 3/7 activity was 322% of control at 24 hours and 859% of control at 48 hours (Fig 1C). MKN45 cells showed a more robust response to 100 nM triptolide treatment with a Caspase 3/7 activation of 1090% of control at 24 hours and 1363% of control at 48 hours (Fig 1D). This indicated that triptolide induced decrease in cell viability stemmed from apoptotic cell death.

### 3.2 Minnelide inhibits growth of subcutaneous xenograft tumors in athymic nude mice

**Fig 2:**
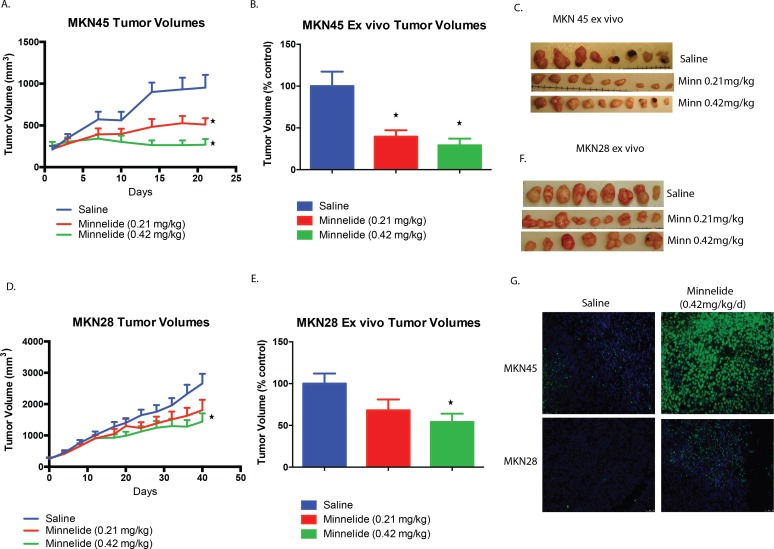
**A. Triptolide prodrug Minnelide induces tumor regression in mouse models for gastric cancer.** Treatment of mice bearing subcutaneous xenograft tumors derived from MKN45 gastric adenocarcinoma cells with Minnelide (0.21 mg/kg) as well as Minnelide (0.42mg/kg) led to a significant reduction in tumor burden compared to control (saline treated) mice. Tumor volumes were assessed twice weekly. Mice were followed for 21 days. **B**. Ex-vivo volumes of the MKN45 derived subcutaneous tumors. **C.** Ex vivo pictures of MKN45 cell derived tumors from mice treated with Minnelide were significantly smaller than the controls. **D.** Treatment of mice bearing subcutaneous xenograft tumors derived from MKN28 gastric adenocarcinoma cells with Minnelide (0.21 mg/kg) as well as Minnelide (0.42mg/kg) led to a reduction in tumor burden compared to control (saline treated) mice. Tumor volumes were assessed twice weekly. Mice were followed for 42 days. **E** Ex-vivo volumes of the MKN28 derived subcutaneous tumors. **F.** Ex vivo pictures of MKN28 cell derived tumors from mice treated with Minnelide were smaller than the controls. **G**. TUNEL staining of MKN45 as well as MKN28 tumors from mice treated with Minnelide showed significantly higher apoptotic cells than the saline treated mice as evidenced by greater numbers of TUNEL positive cells in the Minnelide treated groups. * indicates p value < 0.05 when compared to untreated.

To test the efficacy of the water-soluble pro-drug of triptolide, Minnelide, on gastric cancer cells, we implanted both MKN45 and MKN28 cells subcutaneously in the right flanks of athymic nude mice. Two doses of 0.21 mg/kg/day and 0.42 mg/kg/day were used for treating the animals once the average tumor volumes reached 250 mm^3^. Tumor volume was measured twice weekly to monitor growth. Minnelide treated athymic nude mice had a significantly lower tumor burden than control mice at the end of the experiment for both MKN28 and MKN45 derived tumors. Treatment with 0.21mg/kg/d and 0.42mg/kg/d significantly reduced the tumor burden to 40% and 29% of control for MKN45 derived tumors, respectively (p<0.05) (Fig 2A and 2B). In the MKN28 derived tumor group, 0.42mg/kg/d of Minnelide significantly reduced the tumor burden to 54% of control (p<0.05). (Fig 2C and 2D).

As seen in our in vitro studies, Minnelide treated tumors had a significantly higher proportion of TUNEL positive cells as compared to saline treated tumors, indicating greater DNA fragmentation and apoptosis (Fig 2E). This data indicated that Minnelide treatment significantly slowed tumor progression in vivo by inducing apoptosis.

### 3.3 Minnelide is effective in combination with CPT-11 against gastric cancer xenografts

**Fig 3:**
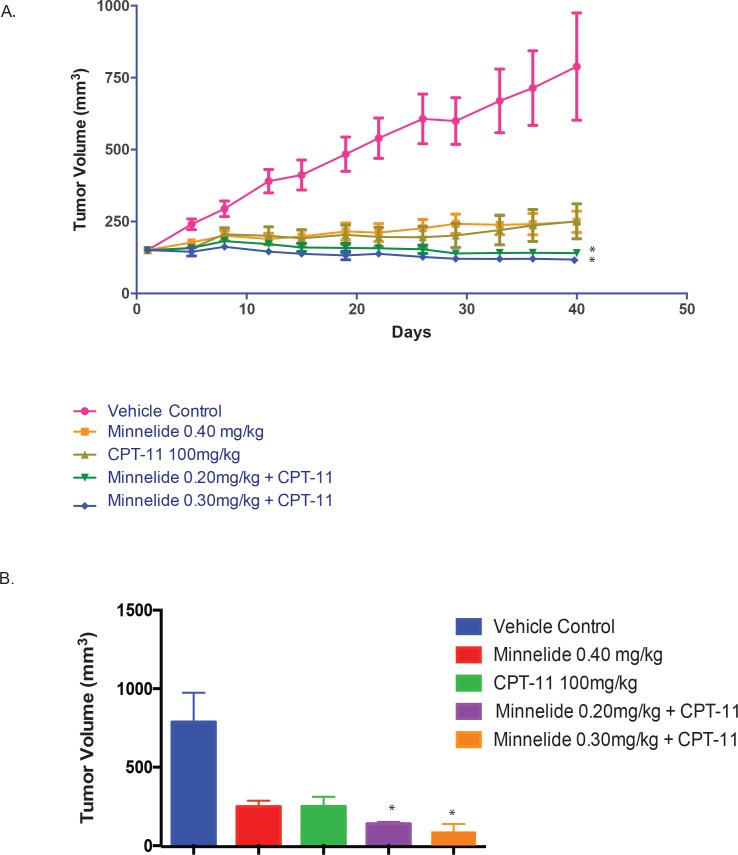
**A. Minnelide and CPT-11 combination is most effective against gastric cancer.** Treatment of mice bearing subcutaneous xenograft tumors derived from NCI-N87 gastric adenocarcinoma cell line with a combination of 0.20mg/kg Minnelide and 100mg/kg CPT-11 or 0.30mg/kg Minnelide and 100mg/kg CPT-11 was more effective in reducing tumor burden as compared to vehicle control or Minnelide 0.40mg/kg monotherapy. **B.** Ex-vivo tumor volumes of the NCI-N87 cell line derived tumors. Combination of lower dose of Minnelide and CPT-11 was more effective in reducing tumor burden compared to vehicle control or Minnelide 0.40 mg/kg alone. * indicates p value < 0.05 when compared to untreated.

To study if Minnelide could be effectively combined with CPT-11, we implanted another gastric cancer cell line NCI-N87 subcutaneously in the flanks of the athymic nude mice, and started treatment after the tumor reached an average volume of 150mm^3^. Treatment was started with Minnelide 0.40 mg/kg, CPT-11 100mg/kg, a combination of CPT-11 and Minnelide 0.30mg/kg and a combination of CPT-11 and Minnelide 0.20mg/kg. Treatment was done for 40 days. The tumor progression and end of study analysis is summarized in Fig 3A and 3B. Our results showed that the tumors responded to the combination of Minnelide and CPT-11 better than the group of Minnelide alone

Treatment with Minnelide 0.20 mg/kg + CPT-11 100 mg/kg resulted in a mean tumor volume of 140.1 mm^3^ by Day 40. This group produced a TGI of 97.5% (n = 4) when compared to the vehicle control on Day 40. This group produced a mean tumor shrinkage of 17.3% (n = 6) on Day 40. A significant decrease in mean tumor volume (p<0.05) was observed when compared to the vehicle control on Day 40. However, no significant difference in mean tumor volume was observed when compared to respective single agent CPT-11. This group experienced mild body weight loss with a maximum of 4.4% on Day 5. Body weights were fully recovered by Day 15.

Treatment with Minnelide 0.30 mg/kg + CPT-11 100 mg/kg resulted in a mean tumor volume of 117.6 mm^3^ by Day 40. This group produced a TGI of 96.4% (n = 2) when compared to the vehicle control on Day 40. This group produced a mean tumor shrinkage of 31.6% (n = 8) on Day 40. A significant decrease in mean tumor volume (p<0.05) was observed when compared to the vehicle control on Day 40. However, no significant difference in mean tumor volume was observed when compared to respective single agent CPT-11. This group experienced moderate body weight loss with a maximum of 5.8% on Day 5. Body weights were fully recovered by Day 15. One of ten mice experienced slight tumor necrosis first observed on Day 5.

### 3.4 Triptolide downregulates Sp1 and HSP70 mRNA in gastric cancer cell lines

**Fig 4:**
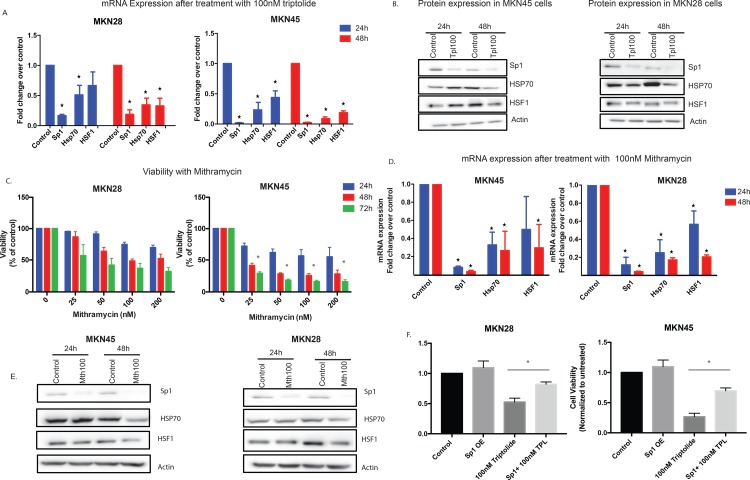
**A Triptolide induced cell death in gastric cancer is mediated via Sp1.** Treatment with 100nM triptolide decreased the mRNA expression of Sp1, HSF1 and HSP70 in MKN28 and MKN45 gastric adenocarcinoma cells in a time (24–48 hours) dependent manner. **B.** Protein expression of Sp1, HSP70 and HSF1 decreased after 100nM triptolide treatment in both MKN28 and MKN45 cells. **C**. Treatment of MKN28 and MKN45 cells with mithramycin (a chemical inhibitor of Sp1) led to a reduction in cell viability in a dose (25–200nM) as well as time (24–72 hours) dependent manner similar to triptolide. **D.** Treatment of MKN28 and MKN45 cells with 100nM mithramycin led to a time (24–48 hours) dependent decrease in the mRNA expression of HSF1 and HSP70, indicating that they lie downstream of Sp1. **E.** Treatment of MKN45 cells with 100nM mithramycin led to a time (24–48 hours) dependent decrease in the protein expression of HSF1 and HSP70, indicating that they lie downstream of Sp1 in gastric adenocarcinoma cells. **F.** Overexpression of Sp1 in gastric cancer cell line MKN28 and MKN45 resulted in a rescue from the triptolide induced cell death. * indicates p value < 0.05 when compared to untreated.

Since Sp1 expression correlated with poor prognosis in gastric cancer, and our previous publication shows that triptolide can downregulate Sp1 leading to a decrease in HSP70 and HSF1, thereby causing cell death (14), we studied the expression of this protein in gastric cancer cells. In gastric cancer cells, triptolide, at a concentration of 100nM decreased the mRNA expression of Sp1 at 24 hours and 48 hours of treatment in both MKN45 and MKN28 cells. Additionally, expression levels of HSF1 and HSP70 were also downregulated (Fig 4A). Consistent with this observation, the expression of Sp1, HSP70 and HSF1 proteins were also downregulated after treatment with triptolide (Fig 4B).

To evaluate whether the loss of viability in the gastric cancer cell lines was indeed due to a reduction in Sp1, we treated cells with a known chemical inhibitor of Sp1, mithramycin (25 -200nM). Treatment with mithramycin reduced the viability of MKN28 cells and MKN45 cells in a dose dependent and time dependent manner, similar to that observed with triptolide (Fig 4C). Further, inhibition of Sp1 (by mithramycin) in gastric cancer cell lines resulted in downregulation of HSF1 and HSP70 expression as well (Fig 4D). Protein expression of HSF1 and HSP70 were also downregulated following Sp1 inhibition by Mithramycin (Fig 4E).

To further confirm that triptolide induced gastric cancer cell death was mediated by inhibition of Sp1, we overexpressed Sp1 in MKN28 and MKN45 cell lines using a pCMV-Sp1 and treated them with 100nM triptolide. Our results showed that overexpression of Sp1 rescued the gastric cancer cells from triptolide induced cell death.

## 4.Discussion

Gastric cancer is the 5^th^ most common cancer in the world. Its prevalence is greatly influenced by geography with the highest incidence being in the republic of Korea, followed by Mongolia and Japan [1]. Gastric cancer is the fifteenth leading cause of cancer related death in the United States [2]. Despite advances in diagnostics, surgical technique and chemotherapeutics, the 5 year survival of gastric cancer patients in the United States is only 29%. This is in part due to the relatively advanced stage of the cancer at the time of diagnosis in the United States, compared to countries like Japan where screening measures are undertaken and consequently more patients are diagnosed with early stage gastric cancer. Over half of the cases of gastric cancer in the western world are stage III or stage IV at presentation [18].

One of the earliest and most widely used classification systems for gastric adenocarcinoma described by Lauren in 1965 describes two distinct histological types: an intestinal type, which is characterized by irregular tubular structures in areas of mucosal inflammation, and a diffuse type, which is characterized by discohesive cells and pools of mucus. In this study we have used two cell lines: the MKN28 cell lines, which falls under the intestinal histological subtype, and the MKN45 which falls under the diffuse histological subtype, according to Laurens classification [19]. Diffuse type carcinomas generally occur in younger patients (age 40–60), have more aggressive behavior and carry a worse prognosis than the intestinal subtype [20].

Depending upon the size and location of the primary tumor, the preferred means of therapy is a total or a subtotal gastrectomy. However with increasing cancer stage, the risk of locoregional relapse increases [21, 22]. Chemotherapeutic options have been evaluated in the neoadjuvant, adjuvant and metastatic settings. The use of adjuvant chemotherapy is estimated to reduce the risk of death by 18% [23]. The MAGIC trial compared surgical intervention along with perioperative chemotherapy (Epirubicin, Cisplatin and 5-Fluorouracil) versus surgery alone and reported a significantly improved 5-year overall survival in the chemotherapy group [24]. Despite the advances in surgery, and the overall improved survival with adjuvant or perioperative chemotherapy, the five year survival of patients with gastric adenocarcinoma in the United States is far less than that of other solid tumors such as breast cancer (5 year survival 89.4%) or prostate cancer (5 year survival 98.9%) (1). This highlights a need for improved chemotherapeutic strategies in addition to surgical resection.

Sp1 (Specificity protein 1) is one of the most well characterized transcriptional activators. It binds to GC rich sequences and is needed for the expression and regulation of a variety of genes, many of which are known to be involved in cancer cell proliferation and survival, evasion of immune destruction, cellular senescence, angiogenesis, tumor cell migration and metastasis [25]. Previous studies have shown that Sp1 is an important regulator of angiogenesis in gastric cancer. It has been demonstrated that treatment of gastric cancer cell lines with mithramycin results in a decrease in the expression of VEGF, a known pro-angiogenic molecule [26]. High Sp1 expression in tumor specimens has been correlated with reduced postoperative survival of gastric cancer patients, possibly indicating a prognostic role of Sp1 [17,27,28]. It has also been reported that early stage gastric cancer is associated with weak or negative Sp1 expression whereas more advanced disease was associated with strong Sp1 expression [28]. Sp1 inhibition has been shown to exert anti-proliferative, anti-migration and anti-invasion effects on gastric cancer cells [29, 30]. We have shown previously in pancreatic cancer, that Sp1 is responsible for the transcription of heat shock factor 1 and heat shock protein 70, both of which are known to have a protective role in cancer cells. Mithramycin is a known chemical inhibitor of Sp1 and we have demonstrated that it reduces cell viability of gastric adenocarcinoma cell lines in a dose and time dependent manner similar to triptolide. We have also demonstrated that reduction in levels of Sp1 using mithramycin are associated with a reduction in HSP70 and HSF1, indicating that Sp1 lies upstream of these pro-survival factors in gastric adenocarcinoma cells (Fig 4).

HSP70 is known to be overexpressed in a variety of human cancers. It has been demonstrated that the overexpression of HSP70 protects cancer cells from apoptosis [31], and is associated with drug resistance and resistance from immune mediated destruction [32]. HSP70 has the ability to inhibit multiple pathways of cell death, including both intrinsic and extrinsic apoptosis, thereby conferring a survival advantage to cancer cells [32, 33]. It has been shown that HSP70 antisense oligonucleotides are able to induce apoptosis and inhibit proliferation of gastric adenocarcinoma cells in a dose and time dependent manner [34]. Studies utilizing resected patient tumor specimens have demonstrated that high HSP70 levels in gastric tumors is associated with poor postoperative overall survival in patients with intestinal type of gastric carcinoma [16]. Our study demonstrates that triptolide treatment results in a reduction of the level of HSP70 in gastric adenocarcinoma cells resulting in apoptosis and cell death.

Our study shows that Minnelide is an effective chemotherapeutic agent for gastric adenocarcinoma. Minnelide is a water soluble prodrug of triptolide which is hydrolyzed to triptolide in the presence of phosphatases once inside the body. Our *in vitro* studies show that triptolide reduces viability of a moderately differentiated intestinal type gastric adenocarcinoma cell line, MKN28 and a poorly differentiated diffuse type gastric adenocarcinoma cell line, MKN45 in a concentration as well as time dependent manner. It acts by inducing apoptosis in the cancer cells as evidenced by caspase activation. It has been shown that triptolide has a variety of inhibitory effects on the pro-survival machinery of many types of cancer cells [35, 36]. Our study indicates that like pancreatic cancer, triptolide inhibits Sp1 in gastric cancer as well to downregulate HSP70 and HSF1 to induce cell death.

Minnelide is currently in clinical trials for GI malignancies. We used Minnelide for the in vivo studies with subcutaneous gastric cancer cell line derived xenograft tumors developed in athymic nude mice. Minnelide was able to effectively decrease the tumor burden at low dose as well as high dose for the MKN45 cell line, while the high dose was effective for the MKN28 cell line. Minnelide treated tumors had significantly greater DNA damage and apoptotic cells as evidenced by TUNEL assay, compared to saline treated tumors (Fig 2). In addition, our results showed that Minnelide worked significantly better in combination with CPT-11 when used in vivo on N87 cell line derived subcutaneous xenografts (Fig 3).

CPT-11 is an alternative name of the anticancer compound Irinotecan. It is a water soluble semi–synthetic derivative of the plant alkaloid camptothecin [37]. Irinotecan has been evaluated for gastric adenocarcinoma both as single agent as well as in combination with other chemotherapies such as cisplatin, mitomycin, or docetaxel [38,39]. Though chemotherapy for advanced gastric adenocarcinoma is still a matter of debate and has regional differences, Guimbaud et al demonstrated that the combination of irinotecan, 5-FU and leucovorin is an acceptable alternative to a platinum based ECX (epirubicin, cisplatin and capecitabine) regimen in first line settings for advanced gastric adenocarcinoma, especially in patients who are unable to receive platinum- based agents [40]. Our study demonstrates that a combination of CPT-11 and Minnelide is an effective therapy for gastric adenocarcinoma and warrants further evaluation by clinical studies.

## 5. Conclusion

We believe that Minnelide could offer a novel and valuable tool for treatment of advanced gastric adenocarcinoma, and further studies to evaluate it as a part of combination chemotherapy are warranted. In the current study, we demonstrate that Minnelide monotherapy as well as in combination with CPT-11 is effective in pre-clinical models of gastric adenocarcinoma, which can be an effective strategy to target gastric cancer in the adjuvant or perioperative setting.

## Abbreviations

Sp1: Specificity protein 1
HSP70: Heat Shock Protein 70
HSF1: Heat shock factor 1
qRT-PCR: quantitative realtime polymerase chain reaction
